# Diphyllin Improves High-Fat Diet-Induced Obesity in Mice Through Brown and Beige Adipocytes

**DOI:** 10.3389/fendo.2020.592818

**Published:** 2020-12-10

**Authors:** Ya-Nan Duan, Xiang Ge, Hao-Wen Jiang, Hong-Jie Zhang, Yu Zhao, Jin-Long Li, Wei Zhang, Jing-Ya Li

**Affiliations:** ^1^ Shanghai Engineering Research Center of Molecular Therapeutics and New Drug Development, School of Chemistry and Molecular Engineering, East China Normal University, Shanghai, China; ^2^ Shanghai Institute of Materia Medica, Chinese Academy of Sciences, Shanghai, China; ^3^ School of Pharmacy, Nantong University, Nantong, China; ^4^ School of Chinese Medicine, Hong Kong Baptist University, Kowloon, Hong Kong Special Administrative Region, People’s Republic of China; ^5^ Kay Laboratory of Brain Functional Genomics, Ministry of Education, Shanghai Key Laboratory of Brain Functional Genomics, School of Life Science, East China Normal University, Shanghai, China

**Keywords:** diphyllin, obesity, brown adipocyte, differentiation, thermogenesis, V-ATPase

## Abstract

Brown adipose tissue (BAT) and beige adipose tissue dissipate metabolic energy and mediate nonshivering thermogenesis, thereby boosting energy expenditure. Increasing the browning of BAT and beige adipose tissue is expected to be a promising strategy for combatting obesity. Through phenotype screening of C3H10-T1/2 mesenchymal stem cells, diphyllin was identified as a promising molecule in promoting brown adipocyte differentiation. *In vitro* studies revealed that diphyllin promoted C3H10-T1/2 cell and primary brown/beige preadipocyte differentiation and thermogenesis, which resulted increased energy consumption. We synthesized the compound and evaluated its effect on metabolism *in vivo*. Chronic experiments revealed that mice fed a high-fat diet (HFD) with 100 mg/kg diphyllin had ameliorated oral glucose tolerance and insulin sensitivity and decreased body weight and fat content ratio. Adaptive thermogenesis in HFD-fed mice under cold stimulation and whole-body energy expenditure were augmented after chronic diphyllin treatment. Diphyllin may be involved in regulating the development of brown and beige adipocytes by inhibiting V-ATPase and reducing intracellular autophagy. This study provides new clues for the discovery of anti-obesity molecules from natural products.

## Introduction

Obesity has reached epidemic proportions worldwide and is associated with an increased risk of metabolic, cardiovascular and chronic inflammatory diseases, such as type 2 diabetes, dyslipidemia, nonalcoholic fatty liver disease (NAFLD), hypertension, coronary heart disease and stroke (1). The fundamental cause of obesity is the chronic energy imbalance between caloric intake and consumption, where surplus energy is stored in adipose tissue (2). Dietary and lifestyle modifications can be effective for the treatment of obesity and the prevention of diabetes. However, these approaches are difficult to maintain in the long term. Efficient ways to increase energy expenditure are urgently needed to combat the escalating occurrence of obesity.

Adipose tissue is a key organ in the regulation of energy balance. Three functionally different types of adipose tissue are present in mammals: white adipose tissue (WAT), brown adipose tissue (BAT) and brown-like (beige) adipose tissue. White adipose tissue is the primary site of triglyceride storage, but BAT and beige adipose tissue are specialized in energy expenditure and adaptational thermogenesis (2). These three tissues differ in anatomical localization, abundance, maintenance throughout the life of the animal, morphology and mainly function (3). The functions of BAT and beige adipose tissue are mediated by uncoupling proteins (UCPs), which are located in the inner mitochondrial membrane. UCP1 uncouples the oxidative phosphorylation process, oxidizing fatty acids and glucose that are produced by triglyceride hydrolysis to generate heat (4). Thus, promoting brown/beige adipose browning is now considered a safe strategy to resist obesity and ameliorate metabolic syndromes, such as hyperglycemia and insulin resistance (5). Strategies to target the browning of brown/beige adipocytes can be divided into two directions according to the characteristics of brown/beige adipocytes: promoting brown/beige preadipocyte differentiation and increasing thermogenesis of brown/beige fat cells (6). After several decades of research, many transcriptional and epigenetic factors and small molecules have been reported to control brown adipose cell fate and function; these factors include PPARγ (7, 8), PRDM16 (9–12) and PGC1α (13–17), and external cues include PPARγ agonists (18), catecholamines, and polyphenols (19).

Natural products are major sources of new drug leads. Searching for molecules with medicinal value from traditional medicines and discovering new indications of bioactive natural products are important approaches in natural product research. Recently, through *in vitro* screening of compounds from natural sources that regulate energy balance, a variety of phytochemicals [e.g., resveratrol (20), quercetin (21), cyanidin-3-glucoside (22, 23), berberine (24), and gypenosides (25)] have been reported to be able to increase energy expenditure and curb obesity and its complications by promoting adipose tissue differentiation and/or thermogenesis capacity.

Aryl naphthalene lignans (ANLs) are widely distributed in dietary or medicinal plants and exhibit excellent biological activities, such as antibacterial, anti-inflammatory, antiviral and anticancer activities (26). In addition, some herbs with ANLs were used as folk medicine for adjuvant therapy of diabetes, such as *Cordia rufescens* (27, 28). Researchers of *Shionogi* & *Co., Ltd.* reported that S-8921 and S-8921G, two synthetic ANLs, exhibited significant hypocholesterolemic effects *in vivo*, which expanded the application of lignans to metabolic diseases (29). Inspired by the potential effects of these ANLs on glycolipid metabolism, an *in vitro* assay based on the effect of browning differentiation in C3H10-T1/2 mesenchymal stem cells were used to evaluate the glycolipid metabolism regulation effects of the ANLs in our natural product library. Interestingly, diphyllin, a common ANL, significantly promoted browning adipogenesis and thermogenesis in adipocytes and increased oxygen consumption under a nontoxic dosage in both C3H10-T1/2 mesenchymal stem cells and primary brown/beige adipocytes. Further investigations revealed that diphyllin reduced obesity and improved the glucose tolerance capacity in high-fat diet-fed C57BL/6J mice. There is less experimental research concerning the effects of these ANLs on glycolipid metabolism. This study may expand new applications of these lignans in metabolic diseases and provide an opportunity to develop a new class of agent against obesity or related diseases.

## Materials and Methods

### Materials

The antibody sources were as follows: UCP-1 (Abclonal, A5857), PRDM16 (Abcam, ab106410), PGC-1α (Calbiochem, ST1202), β-actin (Abgent, AM1021B), Cide-A (Santa Cruz, sc-293289), PPARγ (Abcam, ab178860), adiponectin (AdipoQ) (Abcam, ab62551), FABP4 (Cell Signaling, #2120), LC3 (Proteintech, 14600-1-AP), and p62 (SQSTM1) (MBL, PM045). Rosiglitazone, dexamethasone, 3-Isobutyl-1-methylxanthine (IBMX), 3,3′,5-Triiodo-L-thyronine (T3), indomycine, oligomycin, carbonyl cyanide 4-(trifluoromethoxy) phenylhydrazone (FCCP), rotenone and antimycin A were purchased from Sigma-Aldrich. Recombinant human Insulin (Roche) was purchased from Changzheng Hospital (Shanghai, China). ELISA kits used in measurement of plasma parameters are as follows: TG (Shanghai Fosun Long March, 1.02.1801), TC (Shanghai Fosun Long March, 1.02.0401), NEFA (WAKO, 294-63601).

### Chemical Synthesis of Diphyllin (1)

Diphyllin was prepared under the aldol reaction and Diels-Alder reaction (30). A schematic illustration of the synthesis of diphyllin is summarized in **Scheme S1**. Taking 3,4-dimethoxybenzaldehyde as a starting material, compound 2 was obtained after bromination. Glycolytic acetal 3 was synthesized from 2 and glycol by using p-TsOH as a catalyst in toluene under reflux. Under the condition of n-BuLi, the aldol reaction occurred with piperonal to obtain intermediate 3a, which was further dissolved in glacial acetic acid and reacted with diethyl acetylenedicarboxylate (DEADC) at 140°C under a nitrogen atmosphere to yield 4. The selective reduction of 4 by BH3.Me2S in THF led to diphyllin (**Scheme S1**, **Figures S1–S5**, Supporting Information). The synthesis route was started with 3,4-dimethoxybenzaldehyde under bromination, aldol reaction, Diels-Alder reaction and selective reduction to obtain the target molecule.

*6-Bromoveratrol (2).* Br2 (11.26 ml, 220 mmol) was added to a solution of 3,4-dimethoxybenzaldehyde (33.2 g, 200 mmol) in MeOH (160 ml) in an ice bath over 1 h, and the mixture was stirred for 6 h at room temperature. After the completion of the reaction, as indicated by TLC, 300 ml of water was added, and the crystals were precipitated. To a suspension of the crystal was added aqueous NaOH to adjust it to a pH of approximately 9~10. The precipitate was collected by filtration, washed with water and dried under vacuum to give 49.1 g of 6-bromoveratrol (2) as a white solid (95%). 1H NMR (400 MHz, CDCl_3_) δ 10.18 (1H, s), 7.41 (1H, s), 7.05 (1H, s), 3.96 (3H, s), 3.92 (3H, s).

*2-(2-Bromo-4,5-dimethoxyphenyl)-1,3-dioxolane (3)*. To a flask containing dry toluene (150 ml), 6-bromoveratrol (2) (9.8 g, 40 mmol), glycol (6.5 ml, 48 mmol), and p-toluenesulfonic acid (0.6 g) were added as catalysts. The flask was connected to a Dean-Stark trap and refluxed for 3 h. The solvent was removed in vacuo, and the residue was partitioned between EA and 1 M NaOH (150 ml). The organic layer was washed with brine (250 ml) and dried over anhydrous Na_2_SO_4_. After the solvent was removed, the residue was recrystallized in EtOH to obtain 2-(2-bromo-4,5-dimethoxyphenyl)-1,3-dioxolane (3) (10.2 g, 89%). 1H NMR (400 MHz, CDCl_3_) δ 7.11 (1H, s), 7.01 (1H, s), 5.99 (1H, s), 4.17 (2H, m), 4.06 (2H, m), 3.89 (3H, s), 3.88 (3H, s).

*Diethyl 1-(3,4-methylenedioxyphenyl)-4-hydroxy-6,7-dimethoxynaphthalene-2,3-dicarboxylate (4)*. To a solution of the acetal (3) (2.9 g, 10 mmol) in dry THF (40 ml) was added n-BuLi (4.8 ml, 2.5 M solution in n-hexane, 12 mmol) dropwise at -78°C under N_2_ atmosphere, and the mixture was stirred for 15 min. A solution of piperonal (1.5 g, 10 mmol) in anhydrous THF (10 ml) was added at -78°C. The mixture was stirred for 30 min and allowed to warm to rt for 2.5 h. The reaction was quenched with 50 ml of acetic acid below 0°C. DEADC (1.7 g, 10 mmol) was added to the solution and then heated at 140°C for 1 h in a N_2_ atmosphere. After cooling to room temperature, the mixture was extracted with DCM. The organic layer was washed with brine 3 times and dried over anhydrous Na_2_SO_4_. The residue was purified by silica gel chromatography and eluted with PE/EA = 3:1 to afford 3.01 g of compound 4 as a yellow powder (64%). 1H NMR (400 MHz, Chloroform-d) δ 12.45 (1H, s), 7.74 (1H, s), 6.89 (1H, d, J = 7.8 Hz), 6.81 (1H, d, J = 1.6 Hz), 6.78 (1H, dd, J = 7.8, 1.6 Hz), 6.75 (1H, s), 6.06 (1H, d, J = 1.3 Hz), 6.01 (1H, d, J = 1.3 Hz, 1H), 4.41 (2H, q, J = 7.1 Hz), 4.06 (5H, m), 3.77 (3H, s), 1.36 (3H, t, J = 7.1 Hz), 1.06 (3H, t, J = 7.2 Hz).

*Diphyllin (1)*. To a solution of compound 4 (3.0 g, 6.4 mmol) in THF (40 ml) was added BH_3_·Me_2_S (6.4 ml, 10.0 M solution in DMS, 64 mmol) in an ice bath, and the mixture was stirred overnight at room temperature. After the completion of the reaction, as indicated by TLC, 2 N HCl was added to the solution to adjust the pH to 2~3. Then, the solution was partitioned with DCM. The organic layer was washed with brine (250 ml) and dried over anhydrous Na_2_SO_4_. After the solvent was removed, the residue was recrystallized in MeOH to afford 0.866 g of diphyllin (1) as a yellow powder (36%). 1H NMR (400 MHz, DMSO-d6) δ 10.40 (1H, s), 7.62 (1H, s), 7.02 (1H, d, J = 7.9 Hz), 6.96 (1H, s), 6.86 (1H, d, J = 1.6 Hz), 6.75 (1H, dd, J = 7.9, 1.6 Hz), 6.11 (2H, s), 5.36 (2H, s), 3.94 (3H, s), 3.65 (3H, s). 13C NMR (100 MHz, DMSO-d6) δ 169.7, 150.5, 149.7, 146.8, 146.6, 144.9, 129.5, 129.5, 128.8, 123.8, 123.3, 121.7, 118.7, 111.1, 107.9, 105.4, 101.0, 100.7, 66.6, 55.6, 55.1.

### Chemical Screening

Natural compounds were from Jing-Long Li laboratory, 36 molecules with different skeleton types of natural products (such as lignans, terpenoids, and alkaloids) were selected to treat the cells. For high-throughput chemical screenings, C3H10-T1/2 cells were differentiated in 48-well plates. At day 0 after 2 days of confluency, cells were treated with chemicals for 8 days with induction medium and maintenance medium and analyzed for mRNA levels using quantitative real-time PCR (qRT-PCR) and oil red O staining. The mRNA ratios of *Fapb4*, *Ucp1* and *Adiponectin* in each well were used to evaluate the effect of each chemical, and oil red O staining provided further confirmation. The chemical library was from the Jin-Long Li group and applied at a final concentration of 10 μM or 10 μg/ml of each chemical in screening. Cells treated with DMSO and 1 μM rosiglitazone were used as controls.

### Sulforhodamine B Sodium Salt Assay

Cytotoxicity was determined by the sulforhodamine B sodium salt (SRB) cytotoxicity assay using 96-well microtiter plates as described (31). Cells were plated in duplicate wells (3,000 cells/well) and exposed to diphyllin at different concentrations. After 48 h of incubation, cells were fixed with 10% TCA (attached cells were fixed with 3.3% TCA) solution for 1 h, and 0.057% SRB (Sigma Chemical Co.) was added to each well. After a 30-min incubation, the plates were washed, and dye was dissolved in 10 mM Tris buffer (pH 10.5) and read at 570 nm on a microplate reader (Molecular Device). The wells with cells containing no drugs and wells with medium plus drugs but without cells were used as positive and negative controls, respectively. All of the reagents were purchased from Sigma-Aldrich.

### Cell Culture

C3H10-T1/2 cells were a gift kindly given by Jiqiu Wang, Ruijin Hospital. All the cells were maintained in Dulbecco’s modified Eagle’s medium (DMEM) supplemented with 10% FBS (Gibco), 0.1 mg/ml streptomycin (Invitrogen) and 100 U/ml penicillin at 37°C in a 5% CO2 incubator. Adipocyte differentiation of C3H10-T1/2 cells were induced by treating confluent cells with DMEM containing 10% FBS, 0.5 mM isobutylmethylxanthine (IBMX), 125 nM indomethacin, 1 μM dexamethasone, 850 nM insulin and 1 nM T3. Two days after induction, cells were switched to maintenance medium containing 10% FBS, 850 nM insulin and 1 nM T3 for 6 days. For brown adipocyte differentiation, cells were incubated with diphyllin for 8 days at 5 and 10 μM. For adipocyte thermogenesis, cells were incubated with diphyllin for 24 h after differentiation.

### Isolation of Adipose Stromal Vascular Fraction Cells and Differentiation *In Vitro*

Stromal vascular fraction (SVF) cells were isolated as described from 4- to 6-week-old male C57BL/6J mice previously (32). In brief, adipose tissue from inguinal area and interscapular area was minced on ice and digested with 2.4 mg/ml dispase II (Roche) and 10 mg/ml collagenase D (Roche) in phosphate-buffered saline (PBS) supplemented for 45 min at 37°C, followed by stopping digestion with complete medium and filtering through a 100 μm strainer (BD Biosciences). The cell suspensions were centrifuged at 4 °C for 10 min, 500g, then suspended and filtered through a 40 μm strainer (BD Biosciences) and then further centrifuged at 4 °C for 10 min, 500g, and suspended before plating onto 10 cm dishes. SVF cells were cultured in DMEM/F12 supplemented with 10% FBS and 0.1 mg/ml streptomycin (Invitrogen) and 100 U/ml penicillin (Invitrogen). Adipocyte differentiation was carried out by treating confluent cells in induction medium supplemented with 0.5 mM IBMX, 125 nM indomethacin, 1 μM dexamethasone, 850 nM insulin, and 10 nM T3 for 48 h. Next, the cells were maintained in maintenance medium supplemented with 850 nM insulin and 10 nM T3 for 6 days. To analyze the effects of diphyllin on differentiation, diphyllin was added throughout the differentiation. Experiments were performed on day 8 of differentiation. To analyze the effects of diphyllin on thermogenesis, diphyllin was added at day 7 for 24 h after differentiation.

### Oil Red O Staining

Oil red O staining was performed according to standard protocols (33). Cells were washed twice with phosphate-buffered saline (PBS) and fixed with 4% paraformaldehyde for 30 min at room temperature, followed by oil red O (BBI, E607319) incubation for 60 min. The cells were washed with PBS 2~3 times before visualizing under a light microscope and image capture. Absorbance was measured with a Spectra Max M5 (Agilent Technologies) at 520 nm. The relative intracellular lipid content was expressed as the optical density at 520 nm relative to the solution control.

### Quantitative RT-PCR Analysis

Total RNA was isolated from cells or homogenized tissues using TRIzol reagent (Invitrogen). Complementary DNA was prepared from 1 μg of total RNA using Prime Script Reverse Transcriptase (TaKaRa) according to the manufacturer’s instructions. After 10-fold dilution, the cDNAs were amplified using 2 x SYBR Green qPCR Master Mix (Abclonal) and a Stratagene Mx3005P system (Agilent Technologies). Expression data were normalized to *36b4*. Sequences of primers used in this study are listed in **Table S1** (Supporting Information).

### Western Blot Analysis

Cells were lysed with SDS-PAGE sample loading buffer (Beyotime) on ice and then denatured at 100°C for 10 min. Proteins were electrophoresed through SDS-PAGE and transferred to nitrocellulose filter membranes. Membranes were blocked with 5% skim milk at room temperature for 1 h and incubated with the indicated antibody overnight at 4°C, followed by incubation with secondary antibody at room temperature for 1 h. The expression signal of the indicated proteins was detected with enhanced chemiluminescence (GE Healthcare).

### OCR Measurements

Cells were plated in an XF 96-well microplate (Seahorse Bioscience) and allowed to grow to confluency. The experiments were performed on day 8. For the analysis of diphyllin’s effect on thermogenesis, diphyllin was added at day 7 and for 24 h to C3H10-T1/2 or primary brown/beige adipocytes. The cells were induced to undergo brown/beige adipogenesis for 8 days, followed by OCR measurement at 37°C using an XF96 analyzer (Seahorse Bioscience) according to the manufacturer’s instructions. 2 μM oligomycin, 1 μM FCCP and 1 μM rotenone/antimycin A were used to detect uncoupled respiration, maximal respiration and nonmitochondrial respiration, respectively.

### Chronic Efficacy Studies

All animal experiments were approved by the Animal Care and Use Committee of the Shanghai Institute of Materia Medica, where the experiments were conducted. Six-week-old male C57BL/6J mice (Shanghai SLAC Laboratory Animal Co., Shanghai, China) were housed in a temperature- and relative humidity-controlled room (22 ± 2°C, 55 ± 5%) with a 12-h light-dark cycle and free access to food and water. For chronic anti-obesity studies, mice were fed a high-fat diet (HFD) initiated at 6 weeks of age (60% calories from fat, 20% calories from protein, 20% calories from carbohydrate; Research Diets). At 22 weeks of age, HFD mice were randomly assigned to treatment groups. Vehicle and diphyllin (100 mg/kg) were given between 11:00 and 12:00 AM everyday for 9 weeks. Body weight and food intake were recorded daily. A glucose tolerance test (GTT) was performed in the 4th week. Cold exposure was tested in the 5th week. Metabolic analysis was performed in the 6th week. An insulin tolerance test (ITT) was conducted, and blood samples were collected in the 7th week of the study. Body composition measurements were performed in the 8th week. At the 9th week, tissues were dissected, weighed, immediately frozen in liquid nitrogen and stored at -80°C and fixed with 4% paraformaldehyde at room temperature for HE staining.

### GTT and ITT Assays

The mice were fasted for 6 h and received a *p.o.* injection of glucose (2 g/kg) or *i.p.* injection of recombinant human insulin (0.75 U/kg). The glucose concentrations were measured in blood collected by venous bleeding from the tail vein immediately before and after 15, 30, 60, 90 and 120 min. The area under the curve was calculated for analysis.

### Metabolic Analysis in the Animal Study

Oxygen consumption, carbon dioxide production and locomotor activity were measured using a sixteen-chamber indirect calorimeter (TSE PhenoMaster, TSE system) according to the manufacturer’s instructions. The mice were maintained at 24°C under a 12-h light-dark cycle. Food and water were available ad libitum. Locomotor activity was derived from the x-axis and y-axis beam breaks monitored every 17 min. Heat production as name as energy expenditure, oxygen expenditure, activity and RER were calculated as described previously. The total fat and lean masses of mice were assessed with the body composition in the 8th week of treatment using 1H-nuclear magnetic resonance spectroscopy (Minispec LF90 II, Bruker).

### Cold Exposure

Mice were individually housed at 4°C for 8 h without food but with free access to water. Rectal temperature was measured every hour with a BAT-12 microprobe digital thermometer and RET-3 mouse rectal probe (Physitemp Instruments, Clifton, USA).

### Lysosome Acidification Assays

Lysosomes were acidified using a LysoSensor™ Yellow/Blue DND-160 (Yeasen Biotechnology, 40768ES50) as reported before (34, 35). After diphyllin treatment, cells were incubated for 24 h with 2 μM LysoSensor™ Yellow/Blue DND-160 for 5 min before test. The OD value at 384 nm is used to indicate the pH of lysosomes.

### Histology

Brown adipose tissue, white inguinal and epididymal adipose tissues were fixed in 4% formaldehyde overnight at room temperature, embedded in paraffin, and sectioned (6 μm) by a microtome. The slides were deparaffinized, rehydrated, and stained with hematoxylin and eosin (Sigma) according to a standard protocol. Images were viewed under a light microscope for image capture and cell surface was calculated by AdipoCount application, which was reported before (36).

### Statistics

All the values represent the means ± SEM. Error bars represent SEM; significant differences compared to vehicle controls are indicated by *p < 0.05, **p <0.01, and ***p < 0.001.

## Results

### Diphyllin Is Identified as a Natural Inducer From Differentiation Screening of Natural Products on C3H10-T1/2 Cells

**Figure 1:**
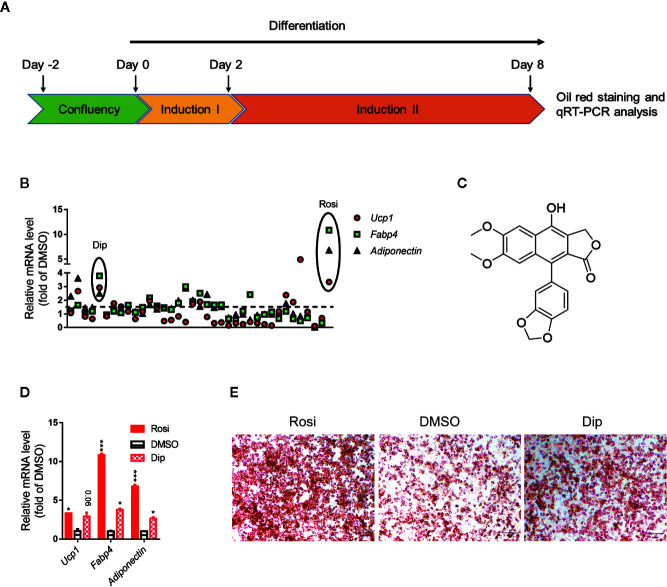
Differentiation screening of natural products in C3H10-T1/2 cells. **(A)** Schematic view of the targeting strategy for natural product screening. **(B)** Relative gene mRNA expression in differentiated cells treated with natural products. **(C)** Structure of diphyllin (1). **(D)** Relative gene mRNA expression in differentiated cells treated with rosiglitazone and diphyllin. **(E)** Oil red O staining in differentiated cells treated with rosiglitazone or diphyllin. For oil red O staining, n=1; for mRNA expression, n=2. *p < 0.05 and ***p < 0.001.

Activation of brown adipocytes contributes to total body fuel expenditure through energy dissipation as heat, which is considered an effective method for the treatment of obesity and related diseases. C3H10-T1/2 cells, which are mouse-derived mesenchymal stem cells, can differentiate into brown adipocytes by a browning cocktail. Based on natural molecules found to promote brown/beige browning, we performed a screening assay on C3H10-T1/2 mesenchymal stem cells, which can be induced into brown adipocytes. For each plate, DMSO and 1 μM rosiglitazone treatment were both included as internal controls. The Jin-long Li natural library was selected for C3H10-T1/2 differentiation, 36 molecules with different skeleton types of natural products (such as lignans, terpenoids, and alkaloids) were selected to treat the cells. Cells were treated with compounds throughout differentiation, and a schematic view of the strategy is shown in **Figure 1A**. The mRNA ratios of *Fapb4*, *Ucp1* and *Adiponectin* in each well were used to evaluate the effect of each chemical, and oil red O staining provided further confirmation. Consistent with many studies, the ratio of rosiglitazone treatment to the control group was significantly differentially promoted with respect to both mRNA (**Figures 1B, D**) and oil red O staining (**Figure 1E**). Fold changes upon treatment with respect to DMSO controls in each plate were then calculated for each compound at the mRNA level (**Figure 1B**). Diphyllin (1) (**Figure 1C**), an aryl naphthalene lignan and a natural compound that can activate *Ucp1*, *Fabp4* and *Adiponectin* expression, showed an mRNA ratio of more than 2-fold (**Figures 1B, D**) and promoted brown adipogenesis, showing more oil red O dye deposition (**Figure 1E**).

### Diphyllin Promotes Differentiation and Thermogenesis of C3H10-T1/2 Cells, Primary Brown and Beige Adipocyte Preadipocytes

The cytotoxicity of diphyllin was initially evaluated in C3H10-T1/2 mesenchymal stem cells with a sulforhodamine B sodium salt (SRB) assay. As shown in **Figure S6** (Supporting Information), at a concentration of 10 μM for 48 h, diphyllin exhibited no apparent cytotoxicity.

**Figure 2:**
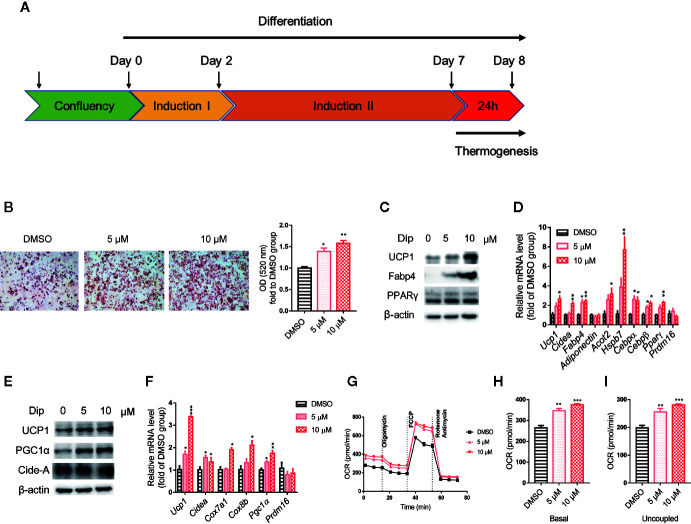
Diphyllin promotes brown adipocyte differentiation and thermogenesis in C3H10-T1/2 cells. **(A)** Schematic view of the targeting strategy to evaluate the effect of diphyllin. **(B)** Oil red O staining in differentiated cells treated with diphyllin, N=3. **(C)** The protein levels in brown adipocytes after the differentiation test; **(D)** The relative gene mRNA levels in brown adipocytes after the differentiation test, n=3. **(E)** The protein levels in brown adipocytes after the thermogenesis test; **(F)** The relative gene mRNA levels in brown adipocytes after the thermogenesis test, n=3. **(G)** Oxygen consumption rates (OCR) of brown adipocytes after 5 and 10 μM diphyllin treatment for 24 h; **(H, I)**. The OCR-related basal metabolic rate and uncoupled oxygen consumption refer to **(C)**. *p < 0.05, **p < 0.01, and ***p < 0.001.

**Figure 3:**
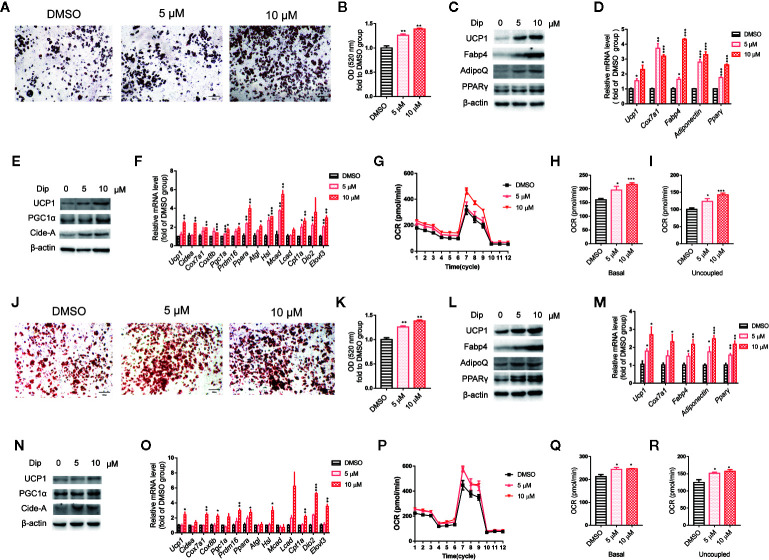
Diphyllin promotes differentiation and thermogenesis in primary brown **(A–I)** and beige **(J–R)** adipocytes. **(A, J)** Oil red O staining of differentiated brown and beige adipocytes treated with diphyllin, N=4. **(B, K)** Quantitative statistics of oil red O staining. **(C, L)** The protein levels in brown adipocytes after the differentiation test; **(D, M)** The relative gene mRNA levels in brown adipocytes after the differentiation test, n=3. **(E, N)** The protein levels in brown adipocytes after the thermogenesis test; **(F, O)** The relative gene mRNA levels in brown adipocytes after the thermogenesis test, n=3. **(G, P)** Oxygen consumption rates (OCRs) of brown adipocytes after 5 and 10 μM diphyllin treatment for 24 h; **(H–I, Q–R)**. The OCR-related basal metabolic rate and uncoupled oxygen consumption. *p < 0.05, **p < 0.01, and ***p < 0.001.

Based on the *in vitro* screening experiment, we evaluated the effects of diphyllin on the browning of brown and beige adipocytes, including the differentiation stage and thermogenesis stage, using the stromal vascular fraction cells of brown fat and white inguinal fat, which could be induced to brown and beige adipocyte (37). For the late phase differentiation of brown and beige adipocyte, after the day 6, the brown and beige marker genes, such as *Adiponectin*, *Fabp4*, *Ucp1*, have reached a very high state, which means that adipocyte was mature (38–40). So, we choose day 7 to investigate the role of diphyllin on mature brown and beige fat, major in thermogenesis activation, except for the role of differentiation. The schematic view strategy is shown in **Figure 2A**. As shown in **Figure 2B**, diphyllin significantly promoted the differentiation of brown preadipocytes at concentrations of 5 and 10 μM by oil red O staining, with adipogenic genes, including *Pparγ*, *Fabp4*, *Adiponectin* and *Ucp1*, being significantly increased (**Figures 2C, D**). In addition, the same phenotypes were reproduced in primary brown and beige adipocyte precursors (**Figures 3A–D**, **Figures 3J–M**). The above data suggested that diphyllin promotes the differentiation of brown/beige adipocytes.

We then evaluated the effects of diphyllin on thermogenesis in differentiated C3H10-T1/2 cells. As shown in **Figures 2E, F**, the expression of thermogenic genes such as *Ucp1* and *Pgc1α* was upregulated in the presence of diphyllin at concentrations of 5 and 10 μM for 24 h. Consistently, the related thermogenic proteins, including UCP1, were also increased after treatment with diphyllin in comparison with the control group. In addition, the basal oxygen consumption rate (OCR) and uncoupling oxygen consumption rate were significantly induced in differentiated C3H10-T1/2 brown adipocytes by coincubation with diphyllin for 24 h (**Figures 2G–I**). In addition, the same phenotypes were reproduced in primary mature brown and beige adipocytes (**Figures 3E–I**, **Figures 3N–R**). Collectively, these results indicated that diphyllin increased thermogenesis in brown/beige adipocytes.

### Diphyllin Promotes Weight Loss With Increased Thermogenesis and Improves Metabolism *In Vivo*

**Figure 4:**
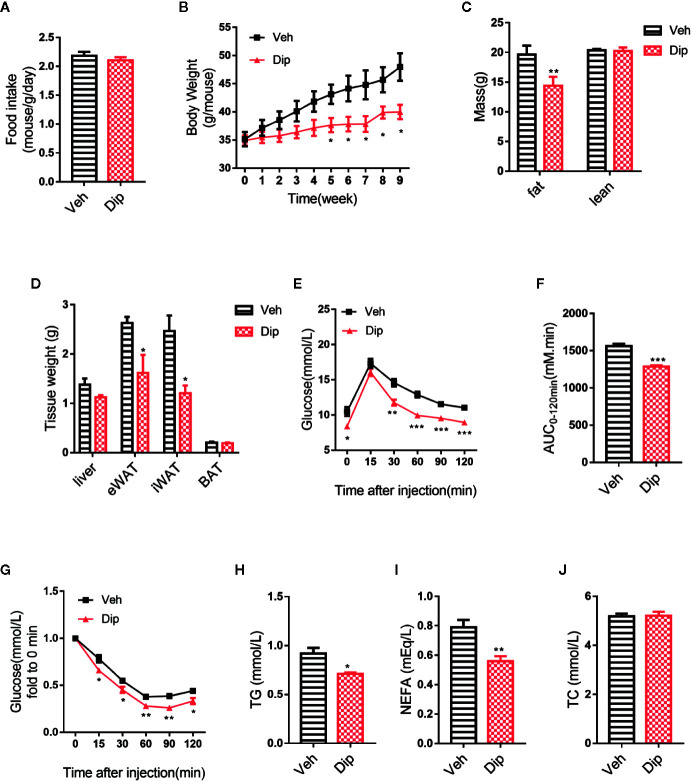
Diphyllin protects mice against high-fat diet-induced obesity. **(A)** Food intake per mouse treated with diphyllin or vehicle. (n = 3). **(B)** Body weights of mice treated with diphyllin or vehicle. (n = 6). **(C)** Lean body mass and fat composition of mice determined by NMR after 5 weeks of treatment. (n = 6). **(D)** Tissue weights of mice determined by NMR after 5 weeks of treatment. (n = 6). **(E, F)** Glucose tolerance test (GTT) performed in mice after 4 weeks of treatment. (n = 6). **(G)** Insulin tolerance test (ITT) performed in mice after 6 weeks of treatment. (n = 6). **(H, I, J)** Plasma total triglyceride (TG), total cholesterol (TC), and nonesterified fatty acid (NEFA) contents of mice measured after 7 weeks of treatment. (n = 6). *p < 0.05, **p < 0.01, and ***p < 0.001.

On the basis of the aforementioned experiments, diphyllin was further evaluated for its effects on the metabolic characteristics of obese mice. After C57BL/6J mice were fed a high-fat diet (HFD-fed mice) for 16 weeks with insulin resistance and obvious obesity, diphyllin was administered orally once daily at a dose of 100 mg/kg for an additional 9 weeks. As shown in **Figure 4B**, significant weight loss of the mice was observed after 4 weeks of diphyllin treatment compared with the vehicle group, while total food intake did not differ between the two groups (**Figure 4A**). We analyzed the body composition of diphyllin-treated mice by NMR (**Figure 4C**). Compared with the control group, the diphyllin treatment group mice had a significantly reduced fat content ratio. In particular, diphyllin treatment significantly reduced the inguinal and epididymis white adipose tissue weights, which suggested that the decreasing weights of mice were mainly attributed to the lowered level of adiposity, since the weights of interscapular brown adipose tissues were similar between the two groups (**Figure 4D**). Consistent with the body weight change, diphyllin treatment resulted in modest improvement in both the glucose tolerance test (GTT) and insulin sensitivity test (ITT), suggesting improved metabolism (**Figures 4E–G**), and diphyllin was found to reduce plasma levels of free fatty acids and triglyceride but not sterol (**Figures 4H–J**).

**Figure 5:**
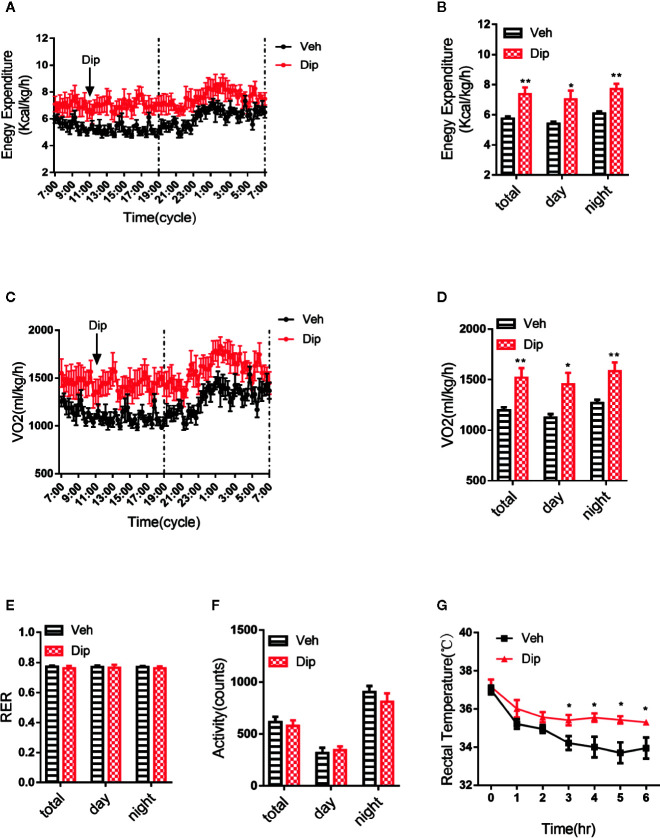
Diphyllin increases energy expenditure and adaptive thermogenesis. **(A)** Energy expenditure and **(B)** statistics for different time periods in DIO mice after 6 weeks of diphyllin treatment (n=6). **(C)** Oxygen consumption (VO2) and **(D)** statistics for different time periods in DIO mice after 6 weeks of diphyllin treatment (n=6). **(E)** Statistics of the respiratory exchange ratio (RER) in DIO mice after 6 weeks of diphyllin treatment (n=6). **(F)** Statistics of the locomotor activity in DIO mice after 6 weeks of diphyllin treatment (n=6). **(G)** Rectal temperature during cold exposure was recorded in 1-h intervals in a cold room (n=6 mice). *p < 0.05 and **p < 0.01.

Energy expenditure is an essential indicator of energy homeostasis, and increasing energy consumption is an important strategy to resist diet-induced obesity. To investigate how diphyllin administration reduced diet-induced obesity, the effects of diphyllin on whole-body energy expenditure were tested with the respiratory metabolic system in HFD-fed mice. After daily treatment for 6 weeks, oxygen consumption and energy expenditure during a 12-h light/dark cycle were higher in diphyllin-treated mice (*p.o.* 100 mg/kg) than control mice (**Figures 5A–D**), with basal respiratory exchange ratio (**Figure 5E**) and unchanged locomotor activity (RER) (**Figure 5F**), which showed that there was no bias in promoting diphyllin between glucose metabolism and lipid metabolism. These results indicated that diphyllin improved diet-induced obesity, which might be attributed to the higher energy expenditure in comparison to control mice. Brown adipose tissue is well known as an essential thermogenic organ that consumes abundant fuels to maintain basic body temperature by nonshivering thermogenesis under cold stimuli, called adaptive thermogenesis. As shown in **Figure 5G**, the mice treated with diphyllin had significantly increased rectal temperature after cold stimulation compared with the control group. All these above data showed that diphyllin treatment could improve glucose and lipid metabolism, resist diet-induced obesity and ameliorate insulin sensitivity.

**Figure 6:**
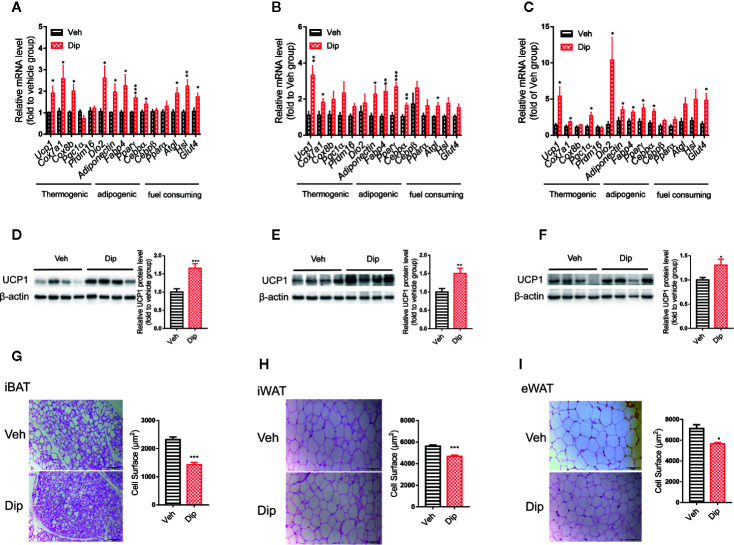
Diphyllin enhances brown/beige thermogenesis and reduces adipocyte size in DIO mice. **(A–C)** Relative mRNA levels of the indicated genes after diphyllin treatment in iBAT, iWAT and eWAT (n=6). **(D–F)** Western blot analysis and relative integrated density of UCP1 protein in iBAT, iWAT, and eWAT of DIO mice after treatment (n=6). **(G–I)** Representative HE staining images and statistical diagram of the cell surface of BAT, iWAT, and eWAT. Scale bar = 100 μm for ×10. *p < 0.05, **p < 0.01, and ***p < 0.001.

Since activation of brown/beige adipocytes is one of the major ways to enhance energy expenditure, we tested whether diphyllin induces reprogramming to brown/beige in adipose tissues. Quantitative real-time PCR revealed that diphyllin upregulated thermogenic genes, including *Ucp1*, *Cox7a1* and *Pgc1α*; adipogenic genes, including *Ap2*, *Adiponectin* and *Pparγ*; and glucose uptake and lipolytic genes, including *Glut4*, *Hsl* and *Atgl*, in inguinal white adipose tissues, epididymal white adipose tissues and interscapular brown adipose tissues (**Figures 6A–C**). The upregulation of *Ucp1* expression in inguinal white adipose, epididymal white adipose and interscapular brown adipose tissues were also confirmed by immunoblotting of UCP1 proteins (**Figures 6D–F**). We also observed that the fat cell surface decreased after diphyllin administration compared to the control group in inguinal white adipose tissues, epididymal white adipose tissues and interscapular brown adipose tissues (**Figures 6G–I**). Based on the above results, we hypothesized that diphyllin augments whole-body energy expenditure to resist obesity by thermogenic activation of adipose tissue in HFD-fed mice.

## Discussion

The worldwide obesity epidemic makes developing a new strategy to combat obesity an urgent need. Few medicines are currently available for the treatment of obesity on the market, and many that are have certain side effects, such as headache, depression and gastrointestinal reaction. Because of its properties, brown/beige adipocyte thermogenesis is considered a safe and effective strategy to combat obesity.

Natural products, owing to their structural and biological activity diversity, represent an important pool for the discovery of drug leads. In our continuing effort to find bioactive molecules that improve adipocyte browning from natural products, we obtained the natural compound diphyllin as a potentiator of brown adipose adipogenesis and thermogenesis by screening our natural product library. Our data showed that diphyllin promoted the thermogenesis and differentiation of brown/beige adipocytes and prevented diet-induced obesity and glycolipid metabolic syndrome in HFD-fed mice. The upregulation of UCP1 in white adipose tissues demonstrated that diphyllin could convert mature white adipocytes into beige adipocytes and promote thermogenesis *in vitro* and *in vivo*.

To determine the diphyllin-induced signaling pathway involved in differentiation and nonshivering thermogenesis, we next investigated the mechanism of diphyllin. Sørensen et al. found that diphyllin, as a novel and naturally potent vacuolar H+-ATPase (V-ATPase) inhibitor, abrogates acidification of the osteoclastic resorption lacunae and bone resorption (41). Diphyllin was previously reported as a V-ATPase inhibitor, which decreases the internal pH (pHi) and reverses the transmembrane pH gradient, inhibits the proliferation and induces the apoptosis of cancer cells and abrogates acidification of the osteoclastic resorption lacunae and bone resorption (42–45). V-ATPase activity as a proton pump is required for acidification of a wide array of different organelles in eukaryotes (46). In mammalian cells, V-ATPase proton pumps are the primary pH regulators that maintain intravascular and/or extracellular pondus hydrogenii (pH). The V-ATPase complex is mainly responsible for lysosomal acidification. Recent reports implicate altered V-ATPase activity and lysosomal pH dysregulation in cellular aging, longevity, and adult-onset neurodegenerative diseases, including forms of Parkinson’s disease and Alzheimer’s disease (47). Ohsumi et al. tested whether vacuolar acidification was required for autophagy in yeast by following the accumulation of autophagic bodies in the vacuoles of wild-type and vma-mutant (V-ATPase deficient) cells and their subsequent degradation. This study confirmed that V-ATPase activity is required for the final step of autophagy, that is, the breakdown of cargo delivered to the vacuole (48). Genetic defects in V-ATPase subunits or V-ATPase-related proteins are increasingly seen in lipid metabolism dysregulation (49, 50) and in association with a decrease in autophagy in the late phase (51, 52). Lysosome-related autophagy regulates brown adipocyte function. Chloroquine, a weak lysosomotropic base that blocks the fusion of autophagosomes with lysosomes, blocked dexamethasone-induced brown adipose tissue whitening and decreased the fat mass content in dexamethasone-treated mice (53, 54). Bafilomycin, the autophagy repressor target of V-ATPase, increases UCP1 protein expression in primary preadipocytes (55). These studies reveal that V-ATPase activity and lysosomes regulate brown/beige cell fate and function.

As inhibiting V-ATPase causes lysosomal deacidification, leading to decreased autophagy flux through autophagosome-lysosome fusion (56), resulting in the accumulation of Microtubule-associated protein 1 light chain 3 (LC3) and sequestosome 1 (p62) (53). We tested the acidification of lysosomes using LysoSensor™ Yellow/Blue DND-160, an acidophilic lysosome shuttle probe, and diphyllin-treated cells showed lower yellow dye deposition than the controls (**Figure S7A**). Autophagy flux was tested with the tandem GFP-RFP-LC3 adenovirus, which represents autophagosome formation as previously described (57). We also observed decreased autophagy flux and an accumulation of LC3 and p62 (**Figures S7B, C**). This suggests that diphyllin may be involved in regulating the development of brown and beige adipocytes by inhibiting V-ATPase and reducing intracellular autophagy.

Autophagy for non-selective bulk degradation of proteins and lipids through the fusion of autophagosomes and lysosomes is suggested as one of the major types of autophagy in adipocytes (58–60). Many studies have shown that autophagy plays an important role in adipogenesis, activation and maintenance of brown/beige adipocytes. An adipocyte-specific mouse knockout of Atg7 increased features of brown adipocytes, which, along with an increase in normal brown adipose tissue, led to an elevated rate of fatty acid beta-oxidation and a lean body mass (60, 61). Inhibition of autophagy in brown adipocytes leads to an increase in UCP1 protein and mitochondrial density, causing more uncoupled respiration and OXPHOS, suggesting a repressing role for autophagy in the activity of the BAT thermogenic machinery (62). Autophagy is needed to convert beige adipocytes to WAT upon removal of β3-AR agonists or recovery from cold exposure, revealing that autophagy plays a negative role in beige adipocyte maintenance (63). It seems that a good anti-obesity strategy would be to inhibit autophagy, but due to inhibition of autophagy seeming detrimental in hypermetabolic conditions such as hepatic steatosis, atherosclerosis, thermal injury, sepsis, and cachexia through an increase in free fatty acid and glycerol release from WAT, the emerging concept of white fat browning–conversion to beige/brown fat has been controversial in its anti-obesity effect through facilitation of weight loss and improvement in metabolic health (64).

In summary, diphyllin improved brown and beige adipocyte differentiation and thermogenesis. Moreover, chronic diphyllin administration alleviated diet-induced obesity by promoting the browning process in brown adipose tissues, inguinal and epididymal white adipose tissues. These results revealed that diphyllin is a promising lead compound for the treatment of obesity and related diseases, and the lead molecule deserves to be further studied.

## Data Availability Statement

The original contributions presented in the study are included in the article/**Supplementary Material**, further inquiries can be directed to the corresponding author/s.

## Ethics Statements

The animal study was reviewed and approved by Animal Care and Use Committee of the Shanghai Institute of Materia Medica.

## Author Contributions

Y-ND and XG contributed to study design, data analyzing, discussion, and preparation of the manuscript. H-WJ, H-JZ, and YZ contributed to conducting the experiments. J-LL, WZ, and J-YL contributed to the study design, discussion, reviewing, and editing the manuscript. All authors contributed to the article and approved the submitted version.

## Funding

This work was supported by the National Natural Science Foundation of China (Nos. 81673493 and 81803384), the National Science and Technology Major Project (No. 2018ZX09711002-018), the Natural Science Foundation of Jiangsu Province, China (No. BK20180947), Strategic Priority Research Program of Chinese Academy of Sciences grant (XDA12050409), Strategic Pilot Program of the Chinese Academy of Sciences (XDA12040204), and the Hong Kong Scholars program (XJ2019054).

## Conflict of Interest

The authors declare that the research was conducted in the absence of any commercial or financial relationships that could be construed as a potential conflict of interest.

## Abbreviations

BAT, brown adipose tissue; Cidea, cell death-inducing DFFA-like effector a; Cox7a1, cytochrome c oxidase polypeptide 7a1; Cox8b, cytochrome c oxidase polypeptide 8b; CPT1, carnitine palmitoyltransferase 1; Dio2, type II iodothyronine deiodinase; EE, energy expenditure; Elovl3, elongation of very long chain fatty acids protein 3; eWAT, epididymal white adipose tissue; FBG, fasting blood glucose; FCCP, carbonyl cyanide 4-(trifluoromethoxy) phenylhydrazone; H&E, hematoxylin and eosin; HFD, High-fat-diet; IBMX, 3-Isobutyl-1-methylxanthine; ITT, insulin tolerance test; iWAT, inguinal white adipose tissue; LC3, Microtubule-associated protein 1 light chain 3; NEFA, nonesterified fatty acid; NMR, 1H-nuclear magnetic resonance; OCR, oxygen consumption rates; P62, sequestosome 1; pH:pondus hydrogeni; PGC-1α, Peroxisome proliferator-activated receptor gamma coactivator 1-alpha; PRDM16, PR domain containing 16; RER, respiratory exchange rate; SVF, stromal vascular fraction; T3, 3,3′,5-Triiodo-L-thyronine; TC, total cholesterol; TG, triglyceride; UCP1, uncoupling protein 1; V-ATPase, vacuolar H^+^-ATPase.
