# Supplementary material for: Diphyllin Improves High-Fat Diet-Induced Obesity in Mice Through Brown and Beige Adipocytes
Source: Front Endocrinol (Lausanne). 2020 Dec 10;11:592818. doi: 10.3389/fendo.2020.592818 (PMC7793827; doi:10.3389/fendo.2020.592818)
Supplement: Supplementary file 1 [file DataSheet_1.docx]

Supplementary Material

# Supplementary Data

**Contents:**

**Figure S1** ^1^H-NMR spectrum of compound **2**. S2

**Figure S2** ^1^H-NMR spectrum of compound **3**. S2

**Figure S3** ^1^H-NMR spectrum of compound **4**. S3

**Figure S4** ^1^H-NMR spectrum of diphyllin (**1**). S3

**Figure S5** ^13^C-NMR spectrum of diphyllin (**1**). S4

**Figure S6** Cytotoxicity of diphyllin in C3H10-T1/2 cell lines.

S4

**Figure S7** Diphyllin reduces intracellular autophagy.

S5

**Table S1** Primer sequences of genes. S6

**Scheme S1** Chemical synthesis of diphyllin (1)*^a^*.

S6


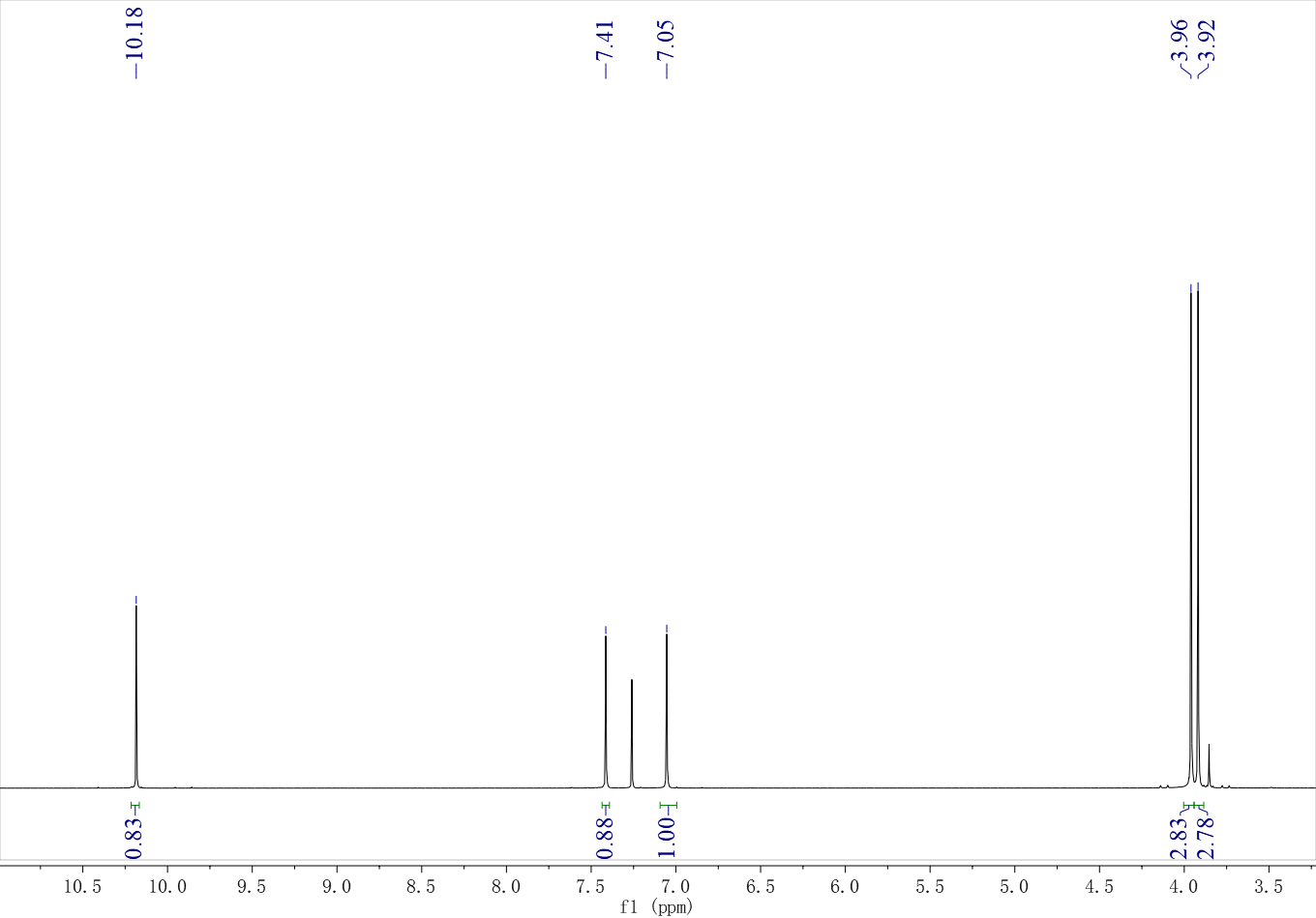


**Figure S1** ^1^H-NMR spectrum of compound **2**

**Figure S2** ^1^H-NMR spectrum of compound **3**

**
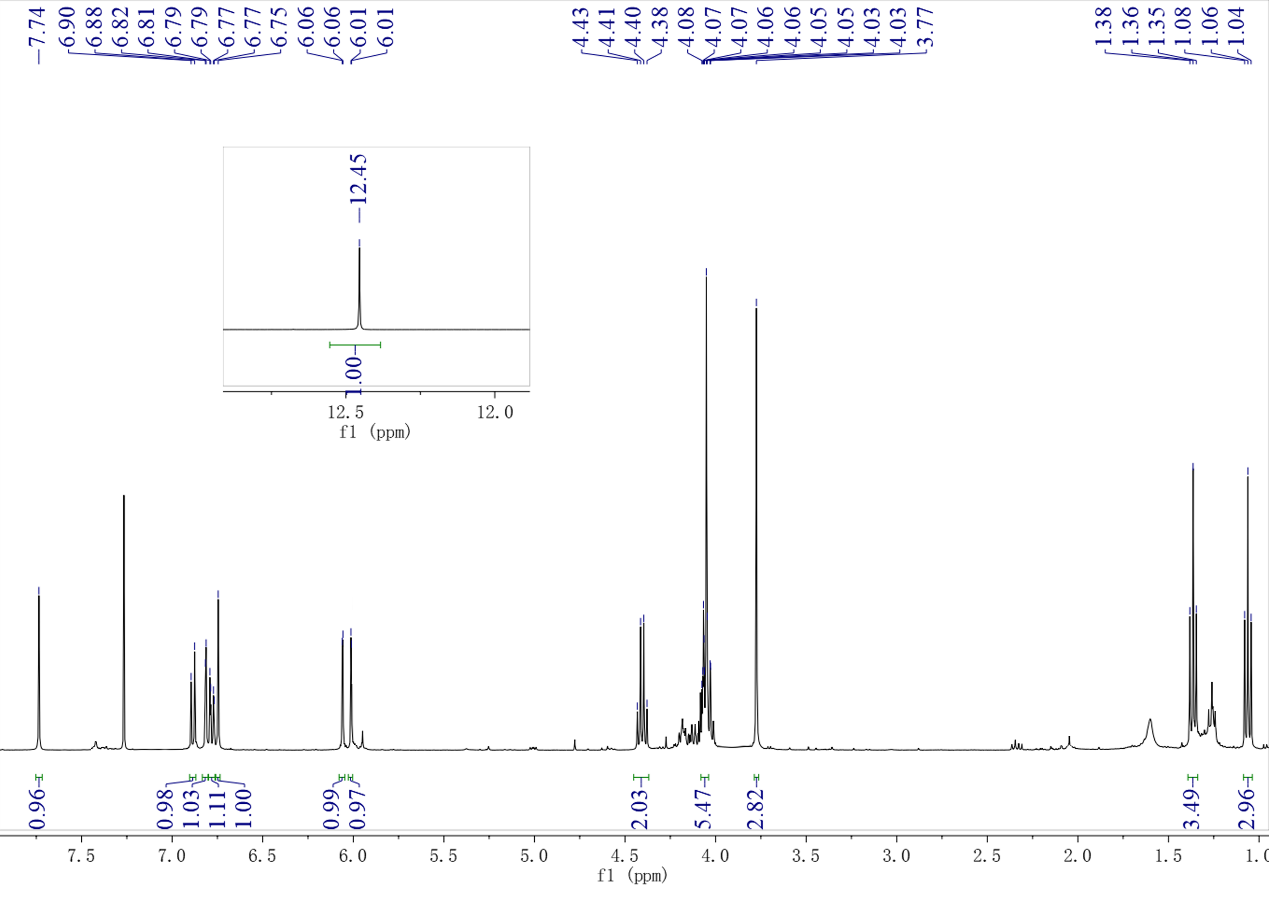
**

**Figure S3** ^1^H-NMR spectrum of compound **4**

**Figure S4** ^1^H-NMR spectrum of diphyllin (**1**)

**Figure S5** ^13^C-NMR spectrum of diphyllin (**1**)

**
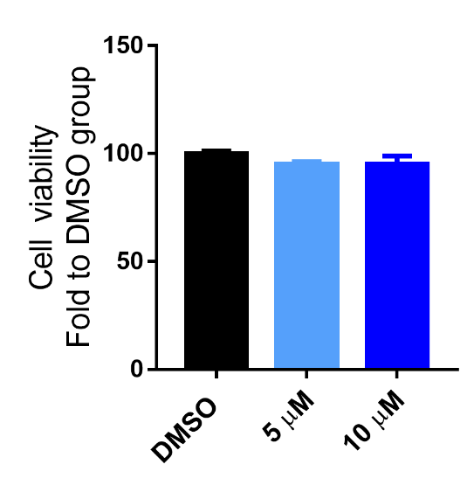
**

**Figure S6** Cytotoxicity of diphyllin in C3H10-T1/2 cells.


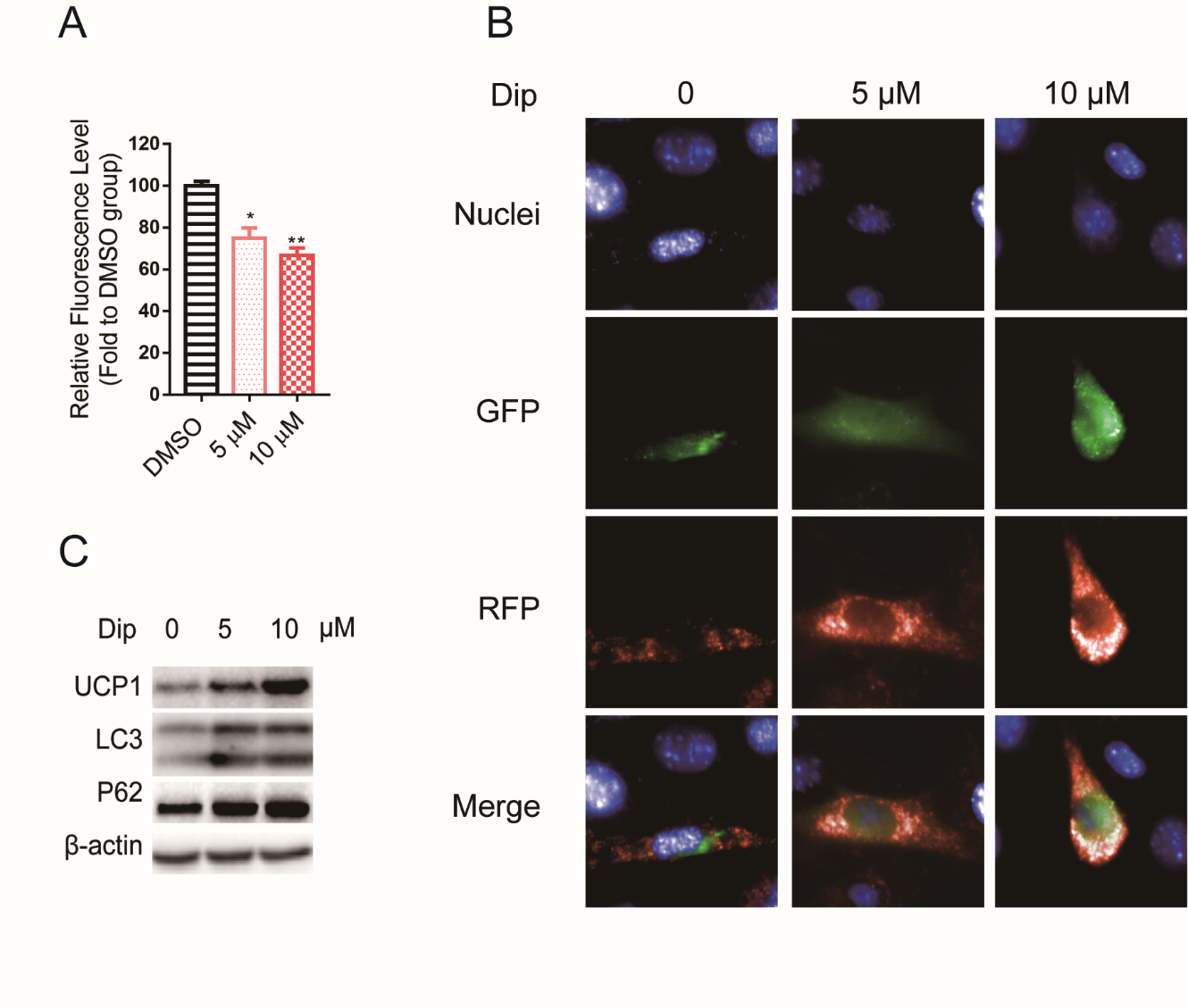


**Figure S7** Diphyllin reduces intracellular autophagy. (A) Relative yellow fluorescence of the lyso sensor DND-160 with DMSO or diphyllin treatment. (B) Image of autophagy flux with DMSO or diphyllin treatment. (C) Effect of LC3 and P62 with DMSO or diphyllin treatment.

**Table S1** Primer sequences of genes.

| Gene | Forward 5’-3’ | Reverse 5’-3’ |
| --- | --- | --- |
| *Ucp1* | ACTGCCACACCTCCAGTCATT | CTTTGCCTCACTCAGGATTGG |
| *Prdm16* | CAGCACGGTGAAGCCATTC | GCGTGCATCCGCTTGTG |
| *Cidea* | TGCTCTTCTGTATCGCCCAGT | GCCGTGTTAAGGAATCTGCTG |
| *Pgc1α* | TATGGAGTGACATAGAGTGTGCT | CCACTTCAATCCACCCAGAAAG |
| *Cox7a1* | CTCTTCCAGGCCGACAATGA | GCCCAGCCCAAGCAGTATAA |
| *Cox8b* | GAACCATGAAGCCAACGACT | GCGAAGTTCACAGTGGTTCC |
| *Pparγ* | TTCCGAAGAACCATCCGATTG | TGGCATTGTGAGACATCCCCAC |
| *Pparα* | AGGCCGTTGCCACTGTTCAG | AGCCCTCTTCATCCCCAAGC |
| *Cebpα* | AGTACCGGGTACGGCGGGAAC | GCGTGTCCAGTTCACGGCTCA |
| *Cebpβ* | TCGGGACTTGATGCAATCC | AAACATCAACAACCCCGC |
| *Fabp4* | ACACCGAGATTTCCTTCAAACTG | CCATCTAGGGTTATGATGCTCTTCA |
| *Acot2* | ATGGTGGCCTCGTCTTTCG | GAGCGGCGGAGGTACAAAC |
| *Hspb7* | GAGCATGTTTTCAGACGACTTTG | CCGAGGGTCTTGATGTTTCCTT |
| *Zic1* | CTGTTGTGGGAGACACGATG | CCTCTTCTCAGGGCTCACAG |
| *Elovl3* | TTCTCACGCGGGTTAAAAATGG | GAGCAACAGATAGACGACCAC |
| *Cpt1α* | CTCCGCCTGAGCCATGAAG | CACCAGTGATGATGCCATTCT |
| *Atgl* | GGTGACCATCTGCCTTCCAG | TGCAGAAGAGACCCAGCAGT |
| *Hsl* | CCGCTGACTTCCTGCAAGAG | CTGGGTCTATGGCGAATCGG |
| *Mcad* | AGCTGCTAGTGGAGCACCAAG | TCGCCATTTCTGCGAGC |
| *Lcad* | TCACCAACCGTGAAGCTCGA | CCAAAAAGAGGCTAATGCCATG |
| *Dio2* | CAGTGTGGTGCACTGCTCCAATC | TGAACCAAAGTTGACCACCAG |
| *Glut4* | CTGATTCTGCTGCCCTTCTGTCCT | GACATTGGACGCTCTCTCTCCAACTT |
| *Adiponectin* | TGACGACACCAAAAGGGCTC | CACAAGTTCCCTTGGGTGGA |
| *36b4* | GAAACTGCTGCCTCACATCCG | GCTGGCACAGTGACCTCACACG |

**Scheme S1** Chemical Synthesis of Diphyllin (1)*^a^*

*^a^*Conditions: (a) 3,4-dimethoxybenzaldehyde (33.2 g, 200 mmol), Br_2_ (11.26 mL, 220 mmol), MeOH, rt, 6 h, 95%; (b) **2** (9.8 g, 40 mmol), glycol (6.5 mL, 48 mmol), p-toluenesulfonic acid (0.6 g, cat.), toluene, reflux, 3 h, 89%; (c) **3** (2.9 g, 10 mmol), n-BuLi (4.8 mL, 2.5 M solution in *n-*hexane, 12 mmol), anhydrous THF, -78 ^o^C to rt, 2.5 h; (d) DEADC (1.7 g, 10 mmol), acetic acid, 140 ^o^C, 1h, 64%; (e) **4** (3.0 g, 6.4 mmol), BH_3_·Me_2_S (6.4 mL, 10.0 M solution in DMS, 64 mmol), THF, rt, overnight, 36%.
